# Recent advances in cell death

**DOI:** 10.1038/s12276-023-01083-0

**Published:** 2023-08-23

**Authors:** Ki-Tae Ha

**Affiliations:** Department of Korean Medical Science, School of Korean Medicine and Korean Medical Research Center for Healthy Aging, Pusan National University, Yangsan, Gyeongnam Republic of Korea

**Keywords:** Apoptosis, Entosis

Death is the irreversible cessation of all biological functions that sustain a living organism, an inevitable end for all living things^1^. No human organ is unimportant, but the three most important in regard to death are the heart, lungs and brain. Traditionally, circulatory-defined death (CCD) has been considered the primary definition of death, although brain death is now widely accepted^2^. No matter which definition of death we use, human cells do not all die at the same time. The cells of a living organism can die before the death of the organism, or they can live on after death and die slowly, having consumed all their oxygen and energy. Cell death is necessary for tissue regeneration, the repair process that delays death and prolongs life^3^. Thus, while forensics were debating when a human being dies, biologists were digging deeper into how cells die.

In the early to mid-19th century, cellular death, also known as necrosis, was reported by Carl Vogt and Rudolf Virchow, among others^4,5^. In 1972, a new type of cell death distinct from necrosis, called apoptosis, was reported by John Kerr^6^. Since then, many other types of cell death have been discovered, and to define them at the molecular level, the Nomenclature Committee on Cell Death (NCCD) revised the “Classification of Cell Death” three times, in 2005, 2009 and 2018^7–9^. The classifications of the NCCD are basically divided into unprogrammed and programmed cell death, with apoptosis, autophagic cell death, lysosomal cell death, pyroptosis, necrotic cell death, NET, etc., being classified as programmed cell death. However, newer cell death types, such as paraptosis, methuosis, alkaliptosis, oxeiptosis, cuproptosis, and erebosis, have been discovered. In addition, immunogenic cell death is still poorly understood, and there is a need to better understand the interactions between different cell death types and the complex forms of cell death. Park et al. documented the historical evolution of various forms of cell death, as described above. They emphasized that understanding the complexity of cell death and its regulatory mechanisms can provide insights into potential therapeutic strategies for various diseases. (Park et al.: 10.1038/s12276-023-01078-x).

Mitochondria, essential double-membrane-bound organelles, govern energy production, support cellular functions, house metabolic pathways, and paradoxically influence cell fate. As a converging point for cell death pathways, mitochondria play a pivotal role in apoptotic and nonapoptotic programmed cell death^4^. Dysfunction of these pathways contributes to age-related conditions such as neurodegenerative, cardiovascular, and metabolic diseases. Strategies targeting mitochondria-associated programmed cell death have promising therapeutic potential, showcasing novel insights in clinical trials^10^. Nguyen et al. focused on mitochondrial quality control networks, their activation by stimuli and their role in maintaining cellular balance via mitohormesis, the mitochondrial unfolded protein response and mitophagy. They also examined different forms of mitochondria-associated programmed cell death, including apoptosis, necroptosis, ferroptosis, pyroptosis, parthanatos and paraptosis, and highlight the involvement of these forms of cell death in the development of age-related diseases. The collective findings highlight potential therapeutic directions for further research. (Nguyen et al.: 10.1038/s12276-023-01046-5).

Recent advances in knowledge of the role of the mitochondria in controlling cell death have heightened interest in the relationship between cell death and cellular metabolism^11^. While it has long been known that increased autophagy due to nutrient deficiencies, such as sugar and amino acid deprivation, is associated with cell death, it is only recently that lipids have been intensively studied as a metabolite involved in cell death^12^. Ferroptosis is a regulated cell death process characterized by iron-dependent lipid peroxidation, contributing to cellular damage in conditions such as cardiovascular diseases, neurodegeneration, liver disease, and cancer^13^. While polyunsaturated fatty acids (PUFAs) are the primary targets for oxidation, saturated/monounsaturated fatty acids (SFAs/MUFAs) also influence lipid peroxidation and ferroptosis. Kim et al. focused on the cellular synthesis of SFA/MUFAs and PUFAs, examining how they are controlled through fatty acid uptake and β-oxidation, which impact susceptibility to ferroptosis. The storage of these fatty acids in various lipids, such as diacyl or ether phospholipids, triglycerides, and cholesterols, and their subsequent release are discussed. By providing an integrated view of these metabolic processes through lipidomic studies, this review contributes insights to the development of therapeutic strategies for ferroptosis-linked diseases, enhancing our understanding of this intricate process. (Kim et al.: 10.1038/s12276-023-01077-y).

The extensively studied regulated cell death (RCD) pathways - pyroptosis, apoptosis, necroptosis, and ferroptosis - play essential roles in maintaining cellular equilibrium, combating infections, and addressing diseases such as cancer^14^. Recent genetic and biochemical investigations have revealed significant flexibility and interplay among these pathways, particularly in response to infections, leading to the concept of PANoptosis^15^. Lee et al. provided a concise overview of these mechanisms, highlighting their significance in cellular balance. The discussion emphasizes the intricate cross-talk among the pathways, focusing on PANoptosis and its relevance in infectious diseases and cancer. A comprehensive understanding of these cell death pathways is crucial for developing innovative therapeutics targeting infections, sterile inflammation, and cancer, and promising advancements in disease treatment. (Lee et al.: 10.1038/s12276-023-01069-y).

Throughout the life cycle of multicellular organisms, cell death occurs via several methods, with apoptosis being the most common under normal conditions. Surprisingly, although the healthy adult human body produces billions of apoptotic cells every day, their existence is rarely detected due to efficient efferocytosis^16,17^. Efferocytosis is a cellular process that removes dead cells and fragments, including pyroptotic and apoptotic cells^7,18^. Over the years, research has focused on unraveling the mechanisms by which phagocytes rapidly and continuously remove apoptotic cells; this process involves intricate molecular and cellular events^17^. Moon et al. discussed the current comprehension of apoptotic cell clearance at the molecular level, shedding light on this essential aspect of cellular maintenance. (Moon et al.: 10.1038/s12276-023-01070-5).

In recent years, there has been an increase in our knowledge of cell death, particularly programmed cell death. However, new types of cell death are likely to be discovered in the future, and the diversity and complexity of cell death will continue to increase. Although this may seem like a disaster for biologists, it will be a great advancement for humanity in terms of increasing the means to prevent and treat disease, aging and ultimately death through a better understanding of the death of the basic unit of life, the cell.
